# Specific emotion regulation difficulties mediate the relationship between personal distress and depressive symptoms in medical students

**DOI:** 10.3389/fpsyg.2024.1432318

**Published:** 2024-08-14

**Authors:** Valentina Colonnello, Paola Castellano, Michela Mazzetti, Paolo Maria Russo

**Affiliations:** Department of Medical and Surgical Sciences, University of Bologna, Bologna, Italy

**Keywords:** personal distress, depression, emotion regulation, mediation, medical students, empathy, health, personality

## Abstract

Several studies indicate a link between personal distress and vulnerability to depression. The literature also suggests that personal distress is associated with emotion dysregulation and that emotion dysregulation plays a role in depression. However, which of the various emotion regulation difficulties mediates the relationship between personal distress and depression remains unexplored. This study therefore aims to investigate the mediating role of specific emotion regulation difficulties in the relationship between personal distress and depression. Of the 702 initially recruited participants, 635 completed a survey comprising the Interpersonal Reactivity Index, the Difficulties in Emotion Regulation Scale, and the Beck Depression Inventory. A mediation analysis was used to explore which emotion regulation difficulties mediate the relationship between personal distress and depression. Over a quarter (27%) of participants reported moderate-severe depression symptoms. Difficulties in accessing adaptive emotion regulation strategies and in having a clear understanding about one's own emotions partly mediated the relationship between personal distress and depression symptoms. Our results are the first to indicate that personal distress is linked to depression risk through specific emotion regulation difficulties in medical students. They also highlight possible modifiable skills that could be targeted by prevention intervention.

## 1 Introduction

The link between personal distress and depression has long intrigued researchers and clinicians. Several studies indicate that depression is mainly linked to impairment in the social domain and in the tendency to experience personal distress (Schreiter et al., 2013; Liu et al., 2022). Personal distress, also called empathic stress, is one of the facets of empathy (Davis, 1983). It has been described as the disposition to feel uncomfortable and experience self-focused negative emotions when viewing another's suffering (Davis, 1983; Decety and Lamm, 2009). Research suggests that personal distress is observed early in infancy, before the development of other-oriented concern, and is influenced by both infant temperament and partly by the quality of parental reactions to children's emotional responses (Eisenberg et al., 2019). Studies have shown that the personal distress trait remains stable over time in non-clinical adult populations (Konrath et al., 2011), is associated with depression (Kim and Han, 2018), and is unaffected by antidepressant treatment (Rütgen et al., 2019).

To understand the intricate relationship between personal distress and depression, it is important to explore the possible mediating mechanisms involved.

Emotion dysregulation is a candidate for mediating this relationship. Both personal distress and depression are characterized in part by difficulties in disengaging from self-focused attention and negative emotions (Beck and Beck, 1972; Davis, 1983), which are expressions of emotion dysregulation. This leads us to wonder whether difficulties in emotion regulation may be a risk factor for depression in individuals with a high personal distress trait.

Literature suggests a link between personal distress and emotion dysregulation (Eisenberg, 1991; Powell, 2018; Ardenghi et al., 2023) as well as between emotion dysregulation and depression (Joormann and Stanton, 2016). Regarding the former, Eisenberg (1991) suggest that, since childhood, a high emotional arousability and a reduced self-regulation play a role in experiencing personal distress when viewing another's suffering. An association between self-reported personal distress and emotion dysregulation has been found both in college students and adults (Okun et al., 2000; Contardi et al., 2016). However, whether personal distress is related to one or more emotion dysregulation domains is unclear.

Emotion dysregulation is a complex phenomenon (Gross, 2001) and, as operationalized by Gratz and Roemer (2004), it involves difficulties in emotional awareness, a lack of clarity about experienced emotions, and a non-acceptance of one's own emotions. It also encompasses difficulties in having access to appropriate emotion regulation strategies, in controlling one's behavior when confronted with negative emotions, and in concentrating and accomplishing tasks during negative emotions.

Regarding the association between emotion dysregulation and depression, research suggests that depressive disorder is related to difficulties in several emotion regulation domains, such as in using adaptive emotion regulation strategies (Joormann and Stanton, 2016; Liu et al., 2022). However, which of the emotion regulation domains as operationalized by Gratz and Roemer mediates the relationship between personal distress and depression remains under investigated.

In sum, while several studies indicate a link between personal distress and emotion dysregulation, as well as between emotion dysregulation and depression, we know of no study that has explicitly disentangled the relationship between these variables. Thus, we aimed to investigate the relationship between these variables and the mediating role of specific emotion dysregulation domains in the relationship between personal distress and depression. Following a developmental perspective, personal distress is viewed as an ontogenetic distal variable that contributes to the risk of developing maladaptive emotion regulation strategies and subsequent psychological outcomes.

We focused on medical students enrolled in their second academic year because this population is at high risk of poor psychological health, with an ~27%−29% prevalence of depression (Bert et al., 2020; Colonnello et al., 2023). Exploring the factors involved in depression symptoms in this population is particularly interesting given that difficulties in emotion regulation strategies have been found to mediate the relationship between individual differences in attachment style and depression in medical students (Colonnello et al., 2022).

## 2 Methods

### 2.1 Participants

Participants were medical students at the beginning of their second academic year of the Medicine and Surgery Degree Program at the University of Bologna's School of Medicine, Italy.

A total of 702 medical students were recruited in two waves of data collection, one in October 2022 and the other in October 2023. They were invited to complete an online survey consisting of questions about sociodemographic characteristics (sex and age) and measures of empathy (Davis, 1983), emotion regulation difficulties (Gratz and Roemer, 2004), and depression (Beck and Beck, 1972). All participants gave informed consent. The procedures were approved by the University of Bologna's institutional review board (IRB no. 273088).

### 2.2. Instruments

Personal distress was measured using the Interpersonal Reactivity Index (IRI, Davis, 1983), which is composed of 28 items and measures: Personal distress, Empathic concern, Fantasy, and Perspective taking. Responses were recorded on a five-point Likert-type scale (0 = Does not describe me very well, 4 = Describes me very well). Cronbach's alpha coefficients ranged between 0.70 and 0.80.

Emotion regulation difficulties were measured using the Difficulties in Emotion Regulation Scale (DERS; Gratz and Roemer, 2004), a 36-item questionnaire that measures six domains of emotion dysregulation: Strategy, Clarity, Goals, Nonacceptance, Impulse control, and Awareness, using a 5-point Likert scale (1 = almost never to 5 = almost always). Cronbach's alpha coefficients ranged between 0.80 and 0.90.

Depression was measured using the 13-item Beck Depression Inventory-Short Form (BDI-SF, Beck and Beck, 1972). The severity of depression is measured on an intensity scale (0–3), with higher scores indicating greater depression. Scores of 0–4 correspond to none or not clinically significant depression, 5–7 to mild depression, 8–15 to moderate depression, and 16 or higher to severe depression. Following Schotte et al. (1997) suggesting of using BDI as a dimensional measure of depression, BDI scores were considered in a continuum. Cronbach's alpha was 0.80.

### 2.3. Statistical analysis

Descriptive statistics were used for categorical and continuous variables. The association between variables was explored using Pearson's correlations. To explore gender differences, we conducted unpaired Student's *t*-tests for personal distress and depression scores, as well as a multivariate analysis of variance (MANOVA) with the DERS subscales as the dependent variables and gender as the independent variable. A multiple regression analysis was performed to test the relationship between personal distress, emotion dysregulation, and depression. Depression was entered as the dependent variable and personal distress and all DERS domains simultaneously entered as predictors, controlling for the factor gender.

Multiple mediation model analyses were conducted using PROCESS macro for SPSS (Model 4, with 5,000 bootstrap resampling), including only the emotion dysregulation domains that correlated with personal distress and depression (Preacher and Hayes, 2008; Hayes, 2013). We computed the total, direct, and indirect path coefficients. Given the studies indicating gender differences in depression risk (Bert et al., 2020), we controlled the mediation model for this factor.

## 3 Results

Of the 702 invited participants, 635 completed the survey (374 women, 261 men, age: *M* = 20.25, SD = 0.96). In the present sample, 27% (*n* = 172) of the participants reported moderate-severe depression (other reported depression levels: without depression symptoms = 52.8%; mild = 20%; moderate = 20.1%; severe = 6.9%). Female participants reported higher personal distress [*t*_(634)_ = 2.708, *p* = 0.007]; however, their depression symptoms were not more severe [*t*_(634)_ = 1.387, *p* = 0.166]. Gender differences emerged in DERS [Wilks λ = 0.97, *F*_(6, 629)_ = 3.34, *p* = 0.003, $\eta_{p}^{2}$ = 0.031]. *Post hoc* analysis revealed that female participants reported higher scores only on the Clarity subscale (see Supplementary Table 1).

Personal distress was related to depression (*r* = 0.35, *p* < 0.01) and to all facets of emotion regulation difficulties except emotional awareness. Depression was associated with all facets of emotion dysregulation, though with different strengths (Supplementary Table 1).

The model including depression as a dependent variable and personal distress and emotion regulation difficulties as independent variables, controlling for gender differences, was significant (*R*^2^ = 0.31, *F* = 41.04, *p* < 0.001). Personal distress (β = 0.171, *p* < 0.01) Strategies (β = 0.365, *p* < 0.01), Clarity (β = 0.119, *p* < 0.01) and Awareness (β = 0.112, *p* < 0.01) were the only significant predictors of depression.

The mediation analysis revealed a significant total effect of personal distress on depression, meaning that the higher the personal distress scores, the more frequent the occurrence of depression symptoms, in the absence of mediators (Table 1).

**Table 1:** Unstandardized (β) and standardized (std β) coefficients of the mediation model (^**^p < 0.001).

**Effect**	**Path**	**β**	**SE**	**95% CI**		**Std ß**
				**Lower**	**Upper**	
Total	Personal distress = > depression (path c)	0.43	0.05	0.34	0.53	0.35^**^
Direct	Personal distress = > depression (path c')	0.23	0.04	0.15	0.32	0.19^**^
Indirect	Personal distress = > strategy = > depression	0.15	0.03	0.10	0.21	0.12^**^
	Personal distress = > clarity = > depression	0.05	0.01	0.02	0.08	0.04^**^
	Personal distress = > impulse = > depression	0.005	0.01	−0.02	0.03	0.004
	Personal distress = > goals = > depression	−0.01	0.02	−0.04	0.03	−0.01
	Personal distress = > nonacceptance = > depression	0.01	0.02	−0.02	0.05	0.01

**Figure 1:**
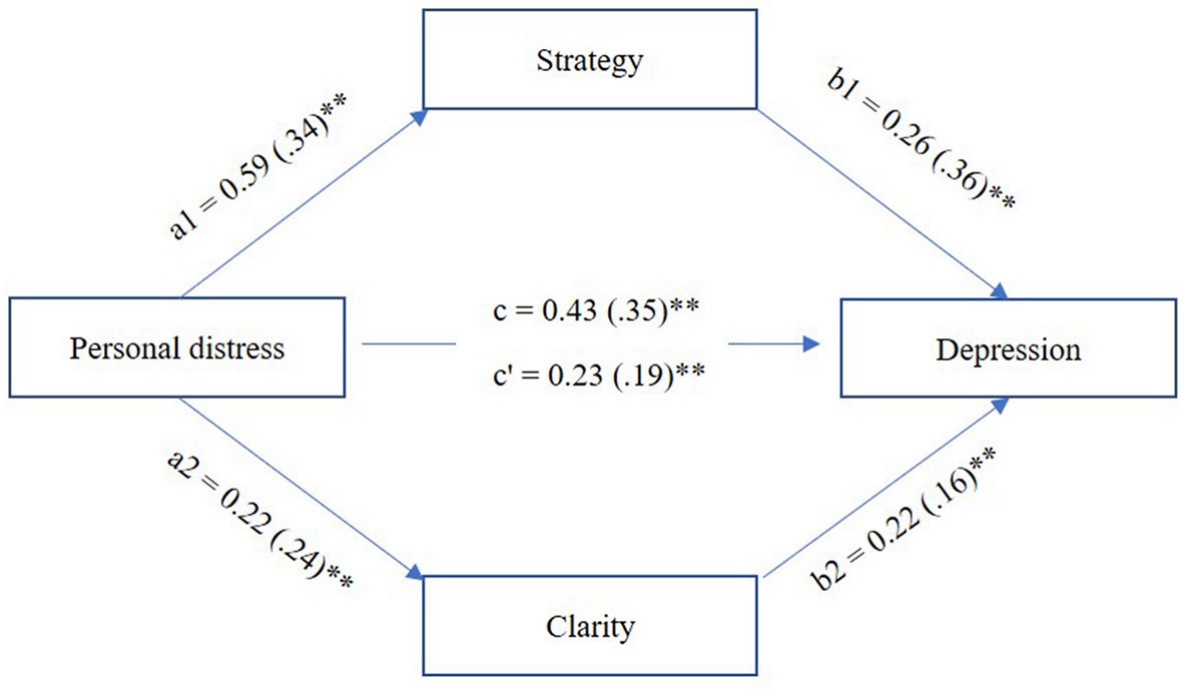
Model of personal distress disposition's mediating effect on depression via strategy (i.e., limited access to adaptive emotion regulation strategies) and clarity (i.e., difficulties in knowing and being clear about one's own emotions). Unstandardized and standardized (in parentheses) path coefficients. **p < 0.001.

This total effect can be divided into direct and indirect effects. The significant direct effect was the effect of personal distress on depression scores when the subscales of difficulties in emotion regulation were considered. For the mediating role of specific emotion regulation difficulties, the indirect effects revealed that Strategies, and marginally Clarity, partly mediated the effect of personal distress on depression (Figure 1).

## 4 Discussion

This is the first study that examines the potential of specific emotion regulation difficulties, based on Gratz and Roemer's operationalization of emotion regulation, in mediating the relationship between personal distress and depression. Despite previous studies having long emphasized the role of personal distress and emotion regulation in the context of depression (Schreiter et al., 2013; Liu et al., 2022), research on their relationship has been limited. The few studies that have explored the role of emotion regulation in the relationship between individual differences in affective empathy and depression have focused on the cognitive processes of emotion dysregulation (Powell, 2018; Liu et al., 2022). Thus, our study represents a novel contribution, as it explicitly explored which emotion regulation difficulties mediate the relationship between personal distress and depression.

Consistent with the existing literature (Joormann and Stanton, 2016; Sendzik et al., 2017; Kim and Han, 2018; Colonnello et al., 2022), our findings indicate that predictors of depression symptoms include personal distress, lack of emotional awareness, poor access to adaptive strategies, and reduced clarity about one's own emotions. Additionally, our findings indicate that lack of emotional awareness, or attention to one's own feelings, is likely not involved in the relationship between personal distress and depression.

Notably, based on correlation analysis, personal distress and emotional awareness were not related. This aligns with literature that conceptualizes personal distress as empathic overarousal and self-oriented feelings of personal discomfort (Davis, 1983; Eisenberg, 1991; Eisenberg et al., 2015), rather than a lack of awareness of experiencing feelings. Our findings suggest that personal distress is related to poor emotion regulation abilities that emerge later in development. These abilities are essential for understanding one's own emotions, distinguishing between one's own and others' emotions, and achieving optimal social functioning.

Among the emotion difficulties related to depression, the only mediating factors were difficulties in accessing emotion regulation strategies and, marginally, difficulties in having clarity about one's own emotions. Thus, individual differences in personal distress play a partial role in the frequency of depression symptoms through specific emotion regulation difficulties.

The mediating role of difficulties in accessing effective emotion regulation strategies is in line with previous literature indicating that depression is associated with the maladaptive use of emotion regulation strategies (Joormann and Stanton, 2016). This finding also adds to the literature indicating that emotion regulation strategies play a role as partial mediator between individual differences in personal distress, that is, in affective dispositions toward interpersonal interactions.

A possible interpretation of this finding is that, in individuals with a high personal distress trait, a heightened negative affective state may limit the cognitive and emotional resources available for identifying and accessing their own effective emotional regulation strategies as well as their learning of how to identify and be clear about their own discrete emotions. This, in turn, could also sustain a chronic dysregulated negative affect, with the consequent negative perceptions of self and self in relation to others that are typical of depression.

Because we found that specific difficulties in emotion regulation only partly mediates the relationship between personal distress and depression, future studies may elucidate the additional factors involved. An interesting focus could be on secure attachment and related comfort and safeness feelings. Research indicates that activation of secure attachment reduces personal distress (Mikulincer et al., 2001). Thus, it would be interesting to investigate the role of secure attachment comfort feelings in accessing regulation strategies and understanding one's own emotions in individuals with a high personal distress trait, and whether these, in turn, reduce depression symptoms.

The current study presents several limitations. First, its cross-sectional nature limits the establishment of causal relationships and may not capture changes in the relationship between variables over time. Although personal distress was considered as a distal variable, the results do not provide insights into how maladaptive emotion regulation strategies and proximal depression symptoms might evolve longitudinally. In addition, the study population was recruited in a single university, which raises questions about the generalizability of the results to broader populations. However, the depression rate in the study population was comparable to rates found in previous studies conducted in several countries (Bert et al., 2020). Thus, our results have implications for medical student populations beyond those in this study.

While cautious interpretation is advised, our findings add to the literature on the role of affective traits in depression risk, revealing for the first time the contribution of specific emotion regulation difficulties in medical students. Our findings corroborate previous studies in medical students documenting personal distress is related to difficulties in emotion regulation and that personal distress is negatively related to problem-oriented coping strategies (Ardenghi et al., 2023, 2024).

Medical students, by virtue of their chosen profession, are likely to be exposed to the suffering and distress of others. Thus, the association we observed between the tendency for personal distress and symptoms of depression underscores that exposing medical students to others' distress may impact their psychological health, depending on individual variations in experiencing distress. The identification of modifiable mechanisms suggests points for intervention, emphasizing the need to develop medical curricula that enhance medical students' understanding of emotional processes (Colonnello, 2021; Colonnello et al., 2022). Integrating training programs that focus on medical students' clarity about their own emotions and accessing adaptive regulation strategies could potentially serve as a preventive measure against the development of depression.

Our study also highlights the necessity for further research to deepen understandings of additional pathways leading to depression. Exploring the role of factors such as attachment styles and self-other distinction can provide a more comprehensive picture of the multifaceted nature of the relationship between personal distress disposition and depression. Notably, although higher depression rates in female students compared to male students have been reported (Bert et al., 2020), our findings showed no significant gender differences. According to Akhtar et al.'s (2020) review on depression in university students, it is possible that barriers to higher education for women are being overcome, thereby reducing gender differences in depression risk. Alternatively, the lack of gender difference in our study might be due to a reduced fear of reporting depression symptoms among male medical students. Supporting this interpretation, a previous study reported no significant gender differences in the fear of specific mental illnesses among medical students (Fino et al., 2019).

This study is of particular significance because it explores whether the effect of personal distress on depression may be mediated by specific emotion regulation difficulties as operationalized in Gratz and Roemer's instrument. By narrowing its focus to medical students, the study fills a critical gap in the literature, offering insights that are particularly relevant to the challenges faced by individuals undergoing medical training.

One notable finding is that not all difficulties in emotion regulation mediate the relationship between personal distress and depression: the contributors to the association between personal distress and depression were traced back to difficulties in accessing effective emotion regulation strategies and difficulties in clarity about one's own emotions. This finding not only refines our understanding of the intricate interplay between personal distress, emotional regulation, and depression but also highlights the key role of potential interventions in enhancing emotion regulation skills.

## Data availability statement

The raw data supporting the conclusions of this article will be made available by the authors, without undue reservation.

## Ethics statement

The studies involving humans were approved by University of Bologna's Institutional Review Board (IRB no. 273088). The studies were conducted in accordance with the local legislation and institutional requirements. The participants provided their written informed consent to participate in this study.

## Author contributions

VC: Conceptualization, Data curation, Formal analysis, Investigation, Methodology, Project administration, Software, Writing – original draft, Writing – review & editing. PC: Writing – review & editing. MM: Writing – review & editing. PR: Writing – original draft.
