# Supplementary material for: Specific emotion regulation difficulties mediate the relationship between personal distress and depressive symptoms in medical students
Source: Front Psychol. 2024 Aug 14;15:1432318. doi: 10.3389/fpsyg.2024.1432318 (PMC11350488; doi:10.3389/fpsyg.2024.1432318)
Supplement: Supplementary file 1 [file Table_1.DOCX]

**Supplementary Materials**

≠

≠

**Table 1.** Correlations between variables (1) IRI_Empathic concern, Interpersonal Reactivity Index, Empathic concern subscale; (2) IRI-Personal Distress, Interpersonal Reactivity Index, Personal Distress subscale; (3) IRI_Perspective Taking, Interpersonal Reactivity index, Perspective Taking subscale; (4) IRI_Fantasy, Interpersonal Reactivity Index, Fantasy subscale; (5) DERS_Non-Acceptance, Difficulties in Emotion Regulation Scale, Non-Acceptance subscale; (6) DERS-Goals, Difficulties in Emotion Regulation Scale, Goals subscale; (7) DERS_Impulse, Difficulties in Emotion Regulation Scale, Impulse control subscale; (8) DERS_Awareness, Difficulties in Emotion Regulation Scale, Awareness subscale; (9) DERS_Strategy, Difficulties in Emotion Regulation Scale, Strategy subscale; (10) DERS_Clarity, Difficulties in Emotion Regulation Scale, Clarity subscale; (11) BDI-Depression, Beck Depression Inventory, depression symptoms. Significant correlations: ⁎ p< .05, ⁎⁎ p < .01; gender differences: ≠ p< .01.
